# An observational study of the occurrence of acute coronary syndrome (ACS) among jordanian patients: Identifying the influence of Ramadan Fasting

**DOI:** 10.1016/j.amsu.2020.09.042

**Published:** 2020-10-02

**Authors:** Liqaa A. Raffee, Khaled Z. Alawneh, Mohammad Khaled Al Suleiman, Rashid K. Ibdah, Sukaina I. Rawashdeh, Abdel-Hameed W. Al-Mistarehi

**Affiliations:** a Department of Accident and Emergency Medicine, Faculty of Medicine, Jordan University of Science and Technology, Irbid, Jordan; b Department of Diagnostic Radiology and Nuclear Medicine, Faculty of Medicine, Jordan University of Science and Technology, Irbid, Jordan; c Division of Cardiology, Department of Internal Medicine, Faculty of Medicine, Jordan University of Science and Technology, Irbid, Jordan; d Department of Public Health and Family Medicine, Faculty of Medicine, Jordan University of Science and Technology, Irbid, Jordan

**Keywords:** Acute coronary disease, Fasting, Jordanian patients ramadan, Kidney function test

## Abstract

**Introduction:**

Patients with Acute Coronary Syndrome (ACS) tend to face several health issues during the Holy month of Ramadan, due to the change in dietary patterns. This study aims to investigate the influence of fasting during Ramadan on the occurrence of ACS.

**Methods:**

The study followed a retrospective observational design, and was conducted in King Abdullah University Hospital (KAUH) of Jordan, during the period of June 06, 2016 to Aug 08, 2016 and May 27, 2017 to July 27, 2017. Data was collected from a sample of 226 male and female patients, aged between 20 and 80 years with major diagnosis of acute coronary syndrome. Therefore, this is a case series of ACS patients.

**Results:**

Findings of the study indicated that, Ramadan fasting is insignificantly related to the occurrence of ACS, since no significant difference was found in the incidence of hypertension (65%), diabetes (51.7%), unstable angina (56.6%) and coronary artery disease (CAD) (57.6%) findings during and after Ramadan respectively. Similar, findings were attained for patients' final diagnosis which had normal Kidney Function Test (KFT) (72.5%), platelets (91.5%), and Ejection Fraction (EF) (64.6%). Also, no significant difference was found between patients’ smoking status (61.0%), hospital stay (89.8%) and discharge rate (96.9%).

**Conclusion:**

The study concluded that there is an insignificant association of Ramadan fasting on the cardiac patients and occurrence of acute coronary syndrome.

## Highlights

• Weak metabolism in patients during fasting causes vascular disease.
• The effect of medicine can be altered by change in eatery habits.
• The findings of the study can support in planning suitable diet for patients with acute coronary syndrome.
• The findings can be practically implemented on extracting new linkage between diet and health conditions.

## Clinicians’ capsule

What is known about the topic?

• Fasting is associated with multiple vascular diseases. The medication's association is reduced due to the significant change in eatery habits.

What did this study ask?

• The study asks for the pattern between the Coronary Artery Diseases (CAD) and Ramadan fasting among Muslims.

What did this study find?

• The study fails to find any significant association of Ramadan fasting on cardiac patients, and thus did not see any relationship of fasting with performing their routine work.

Why does this study matter to clinicians?

• Treatment outcomes of patients provide an explicit insight regarding the significant risk factors of ACS during and after Ramadan.

## 1 Introduction

The ritual of fasting by Muslims is associated to vascular diseases such as; lipid profile and poor glycaemic controls in patients with weak metabolic system. Besides the change in eating patterns causes a significant change in the use and impact of medicines and physiological conditions [[1], [2], [3]]. Turin et al. outlined that, fasting during the month of Ramadan adds a drastic change in an individuals’ life style and health, because of consuming food that is high with fat, carbohydrates, rich in sugar and other ingredients [4].

Patients with acute coronary syndrome (ACS) are identified as those facing complicated health issues [5]. Studies conducted by Alhaddad et al. and Alsheikh-Ali et al. indicated ACS as one of the leading causes of death specifically in Middle East [6,7]. Moreover, it has been identified as considerably responsible for the occurrence of increased deaths due to cardiovascular diseases (CVDs) [8,9]. Acute chest pain serves as the most common symptom in majority patients with ACS [10]. Kishk et al. proposed that the change in diet and sleep timings are significant in affecting the cardiac rhythm and further influences the presentation timings of ACS [11]. Alshareef observed a significant increase in the prevalence of cardiovascular diseases and ACS among different populations, specifically during and after Ramadan [12]. Mousav et al. provided a detailed analysis regarding the effect of fasting on coronary artery disease (CAD) [13]. Findings indicated that patients with CAD were able to observe fast, while symptoms of chest pain were like that in normal conditions.

Problem related to the association of fasting and the rate of ACS has provided contradicting results in most of the studies. Pekdemir et al. identified significant association of the change of dietary and sleeping patterns during the month of Ramadan, with the change in health conditions of patients with cardiovascular diseases [14]. Contrary to this, Mousavi et al. observed no significant change among patients with CAD during and after the month of Ramadan, since the number of patients that were admitted during the given period was like those in normal days [13]. For Chamsi-Pasha et al. individuals exposed to the high-level risks of ACS are advised to exempt fasting to avoid severe illness [15]. One of the basic reasons for increased risk probabilities of ACS is the consumption of calories which is significantly increased due to the altered meal preferences in Ramadan [16]. Meo outlined that in Ramadan, a normal individual consumes 1220 Kcal/day along with the loss of body weight for about 2.0 kg. In addition, several physiological and biochemical changes have been observed during the month of Ramadan that ultimately affect their health [17]. Abazid et al. further pinpointed that for patients with Reduced ejection fraction (HFrEF), fasting is considerably safe, whereas, patients that are not adhered to diet and medication tend to suffer with decompensated heart failure during Ramadan [18].

Considering the contradicting results proposed by previous studies, this study aims to investigate the influence of fasting during Ramadan on the occurrence of ACS in patients admitted in King Abdullah University Hospital (KAUH) with the major complaints of chest pain. A comparative analysis of patients’ diagnosis is undertaken by examining patients that were admitted during and after Ramadan. Contributions of this study are significant, as the information is highly useful for clinical experts in identifying the prevalence of ACS in Ramadan. The study is further important in the identification of risk factors associated to ACS, specifically among Jordanian population during the month of Ramadan.

## 2 Material and methods

### 2.1 Study design and settings

A single centre, retrospective observational study design has been followed. The study is conducted in King Abdullah University Hospital (KAUH) of Jordan. The study period was from June 06, 2016 (1-Ramadan-1437) to Aug 08, 2016 (5- Dhuʻl-Qiʻdah-1437), and May 27, 2017 (1-Ramadan-1438) to July 27, 2017 (4- Dhuʻl-Qiʻdah-1438). An ethical approval regarding the collection of data was obtained from the Institutional Review Board (IRB) of KAUH.

### 2.2 Study population

The population of this study includes, patients with acute coronary syndrome that were admitted during and after Ramadan at the King Abdullah University Hospital (KAUH) of Jordan. Study sample includes, 226 male and female patients aged between 20 and 80 years with major diagnosis of acute coronary syndrome.

### 2.3 Inclusion and exclusion criteria

The inclusion criteria for this study was patients suffering from hypertension, diabetes mellitus, family history of Ischemic heart disease (IHD), hyper/dyslipidaemia, CAD, cardiac arrest, and changes in cardiac enzymes. However, patients less than 20 years, those who did not fast, or pregnant women were excluded from this study. This age group is specified since most of the patients suffering from heart disease were above 20 years [19].

### 2.4 Definitions

#### 2.4.1 Acute coronary syndrome

Acute Coronary Syndrome describe different conditions associated to the reduced flow of blood to the heart. The disease is further associated to the severe discomfort and chest pain [20]. Individuals suffering from myocardial damage due to acute coronary syndrome and elevated ischemic markers are at increased risk of fatality; however, few of the cases are not presented with critical angiography stenosis.

#### 2.4.2 Coronary artery disease

Coronary Artery Disease develops when major blood vessels that are the important suppliers of nutrients, oxygen and blood are damaged or ceased. Arteries are further occupied with cholesterol containing plaque which further narrows arteries resulting in decreased blood flow [21]. At present, invasive coronary angiography is recommended to patients suffering from chest pain, heart failure, or suspected CAD.

#### 2.4.3 Ischemic Heart Disease

Ischemic Heart Disease is caused by the narrowing of heart diseases, resulting in decreased supply of oxygen and blood towards heart muscles. The disease is also referred as, coronary heart disease leading towards heart attack. Coronary angiography is suggested as a clinical diagnostic procedure for confirming IHD, among the patients requiring revascularization.

### 2.5 Data collection and analysis

Data base and medical records of patients that were admitted during the period of June 06, 2016 to Aug 08, 2016 and May 27, 2017 to July 27, 2017 were reviewed. The period specifically included patients that were admitted during Ramadan and after Ramadan. Patients’ reports specifically related to ECG, cardiac enzymes, results of cardiac catheterization and final diagnosis were also followed. Other specific details such as the weather conditions during and after Ramadan were further noted. Where, during the month of May and June there is long exhausting summer and temperature reaches up to 38 °C or above, and fasting lasts for 13–14 h, which is quite long hours for people belonging to the heart disease.

Statistical Package of Social Sciences (SPSS) version 23.0 was used to analyse the findings of the study. Results of the study were indicated through descriptive statistics, and were compliant to STROCSS guidance [22]. However, p-value of less than 0.05 is considered significant.

## 3 Results

To identify the influence of Ramadan fasting on the occurrence of ACS patients, 226 patients were enrolled following the exclusion and inclusion criteria. Table 1 provides the demographic profile of the respondents including their gender and age. Table 2 shows the mean and standard deviation of significant biological markers of the patients. Hypertension shows the highest mean value amongst significant biological markers (M = 2.61) followed by smoking (M = 2.60), previous history of IHD (M = 2.52), diabetes mellitus (M = 2.45). This shows that hypertension was the most prevalent biological marker reported among the patients. Where minimum mean value i.e. 2 represents the second stage of hypertension, whereas maximum mean value (3) represents the crisis stage of the hypertension with systolic 180 or above, and diastolic 120 or higher.

**Table 1:** Demographic characteristics.

Variables	All Patients (226)		During Ramadan		After Ramadan		P-value
	N	%	N	%	N	%	
Gender							
Male	176	77.8%	92	32.5%	84	29.7%	0.014
Female	50	22.1%	20	31.3%	30	46.9%	
Age							0.503
Between 20 and 35 years	9	3.9%	3	23.1%	6	46.2%	
Between 36 and 50 years	69	30.5%	30	28.0%	39	36.4%	
Between 51 and 65 years	91	40.26%	52	35.9%	39	26.9%	
Between 66 and 80 years	57	25.2%	27	30.5%	30	36.6%

**Table 2:** Significant biological markers.

	Minimum	Maximum	Mean	Std. Deviation
Hypertension	2	3	2.61	.490
Diabetes Mellitus	2	3	2.45	.498
Previous history of IHD	2	3	2.52	.500
Family history of IHD	2	3	2.28	.448
Smoking	2	3	2.60	.491
Lipid profile	1	3	2.24	.434

Table 3 then provide results in relation to the final diagnosis of patients during and after Ramadan. Findings indicated that, the majority patients i.e. 57.5% did not report CAD during and after Ramadan, whereas, 72.4% patients reported the normal level of CAD, in both during and after Ramadan. The findings about cardiac arrest were not common among 95% patients that reported during and after Ramadan.

**Table 3:** Final diagnosis of patients during and after ramadan.

Variables	All Patients (226)		During Ramadan		After Ramadan		P-Value
	N	%	N	%	N	%	
CAD							<0.000
Patients with CAD	96	42.4%	48	21.2%	48	21.2%	
Patients without CAD	130	57.5%	64	28.3%	66	29.2%	
Kidney Function Test							<0.000
High	62	27.2%	30	13.2%	32	14.1%	
Normal	164	72.4%	82	36.2%	82	36.2%	
Cardiac arrest							0.634
Yes	11	4.7%	7	3.0%	4	1.7%	
No	215	95%	105	46.4%	110	48.6%	
Platelets (Cut-off below 150 × 10^9^/L)							0.58
High	2	0.8%	0	0.0%	2	0.8%	
Low	17	7.4%	8	3.5%	9	3.9%	
Normal	207	91.5%	104	46.0%	103	45.5%	
Echocardiogram							<0.000
Low EF (41%–49%)	65	28.6%	25	11.0%	40	17.6%	
NA (70%+)	15	6.5%	8	3.5%	7	3.0%	
Normal EF (50%–70%)	146	64.5%	79	34.9%	67	29.6%	
Final Diagnosis (Changes in cardiac enzymes)							<0.000
Non –STEMI	52	22.9%	27	11.9%	25	11.0%	
STEMI	46	20.2%	16	7.0%	30	13.2%	
Unstable Angina	128	56.6%	69	30.5%	59	26.1%	
Hospital Stay							0.480
1–5 days	203	89.7%	101	44.6%	102	45.1%	
6–10 days	20	8.8%	10	4.4%	10	4.4%	
11–15 days	1	33.3%	0	0.0%	1	33.3%	
Above 15 days	2	0.8%	1	0.4%	1	0.4%	
Outcomes							0.933
Discharged	218	96.8%	109	48.2%	110	48.6%	
Dead	7	3.0%	3	1.3%	4	1.7%	

The number of platelets reported by 91.5% patients during and after Ramadan was normal. Also, 64.5% patients who reported during and after Ramadan showed normal Ejection Fraction (EF). Unstable angina was common among 56.6% patients reported during and after Ramadan. 89.7% patients were hospitalized for 1–5 days during and after Ramadan. The overall findings resulted in the discharge of 96.8% patients that were admitted during and after Ramadan. Findings further indicated CAD, KFT, ECG, and changes in cardiac enzymes as significant indictors of ACS among Jordanian patients during and after Ramadan (p < 0.001).

Table 4 shows the comparison of different laboratory investigations during and after Ramadan. Significant difference was only shown for echocardiogram with p-value of 0.05; whereas, there was no significant difference in any of the other modalities. These findings indicate that Ramadan fasting creates an insignificant association on the occurrence of ACS.

**Table 4:** Comparing individuals during and after Ramadan corresponding to different factors.

	Unstandardized Coefficients		Standardized Coefficients	t	Sig.
	B	Std. Error	Beta		
Diabetes Mellitus	0.001	0.100	0.000	0.005	>0.000
Hypertension	0.029	0.109	0.018	0.269	>0.000
Previous history of IHD	−0.018	0.099	−0.011	−0.181	>0.000
Family history of IHD	0.001	0.100	0.000	0.007	>0.000
Lipid profile	−0.046	0.103	−0.024	−0.444	>0.000
Smoking	0.108	0.096	0.066	1.132	>0.000
CAD	−0.011	0.050	−0.013	−0.221	>0.000
Outcome	0.730	0.416	0.159	1.755	>0.000
Echocardiogram	0.093	0.048	0.107	1.923	<0.000
KFT	0.012	0.116	0.006	0.103	>0.000
Platelets	0.125	0.146	0.047	0.856	>0.000

## 4 Discussion

The present study determined the association of fasting on patients with acute coronary syndrome. The study indicated that, patients who reported during and after Ramadan were diagnosed through hypertension, smoking, family history of IHDS and hyper/dyslipidaemia. In addition, the final diagnosis of patients indicated that, most of the patients showed normal KFT (creatine), along with the normal number of platelets during and after Ramadan. Also, most of the patients had normal EF results in the given diagnostic durations. Similarly, in the final diagnosis of patients, unstable angina was common among majority of participants. Overall results indicated no significant association of fasting on the occurrence of ACS.

These findings are similar to those proposed by Salam et al. where, examinations regarding the association of fasting on patients with acute heart failure (AHF) were undertaken [23]. Findings of the study indicated that, patients admitted during the month of Ramadan showed minimal symptoms in relation to the prevalence of AHF, in comparison to those admitted after Ramadan. In addition, the arterial arrhythmias and cholesterol levels were significantly low during Ramadan, indicating that Ramadan fasting was not independently associated to increased prevalence of AHF.

Results proposed by Al-Suwaidi et al. are in line with the findings of the present study [24]. The examinations regarding the occurrence of coronary heart diseases during the 10-year period were undertaken. Findings of the study indicated no significant differences in the occurrence of ACS in three months duration i.e. before Ramadan, during Ramadan and after Ramadan. Similar to the results of the present study, the study indicated no significant difference in relation to the clinical characteristics of patients, including age, sex, hypertension, diabetes, and patients’ death rate.

Al JS, Zubaid et al. conducted another study to determine the association of fasting on patients with heart disease [25]. The general characteristics of patients include: the history of congestive heart failure, angina, atrial fibrillation and prosthetic metallic valves. Findings indicated that most of these patients were able to fast, whereas only few of them felt worst when fasting. Moreover, most of the patients complained for cardiac medications whereas, other felt the need for dietary instructions. However, the study concluded that fasting has no significant association on patients with cardiac disease. These results are in line with those proposed in the present study, where fasting indicated no significant association on the occurrence of ACS.

Kishk et al. conducted a study to determine the association of fasting on the cardiac patterns of acute coronary syndromes (ACS) [11]. The study provided that most of the fasting patients had ACS symptoms between 3 a.m. and 4 a.m. Whereas, non-fasting patients majorly experienced ACS symptoms between 7 a.m. and 8 a.m. Findings of the study therefore, indicated that circadian rhythm is highly influenced through the change in food intake and sleep timings. These factors are further significant in determining the occurrence of ACS symptoms. These findings are in contrast with those proposed in the present study.

Schwartz et al. provided additional information in relation to the fasting triglyceride levels among patients with a history of ACS and treatment of statins [26]. Findings indicated that fasting triglyceride levels were majorly associated to long-and short-term risk factors of ACS. In addition, Steen et al. conducted a study to identify the association of fasting on patients with ACS [27]. Findings indicated that fasting samples had an independent association with high levels of low-density lipoprotein cholesterol (LDL-C) and low levels of triglycerides (TG). The study further concluded that fasting is insignificant in altering the high-density lipoprotein cholesterol, apolipoprotein C-III and total cholesterol.

This study involves certain limitations. The first and the major limitation of the study is that comparative findings of both stable and unstable patients were not explored in this study like those with advanced unstable angina or heart failure. Another limitation of the study is its small sample size due to which the results cannot be generalized. Since the study is based on retrospective data, probabilities in the change of existing conditions is possible if future studies are expanded based on weather conditions and other internal and external factors.

## 5 Conclusion

The results depicted insignificant association of Ramadan fasting on cardiac patients that indicates that patients diagnosed with acute coronary syndrome may observe fasting in Ramadan to fulfil their religious duty. Findings of the study are significant for medical experts, as it identifies treatment outcomes of patients, while providing an in-depth insight about the considerable risk factors of ACS during and after Ramadan. Future researchers are suggested to conduct a study that specifically focuses on the association of patient's diet on ACS occurrence in the month of Ramadan. They are further recommended to conduct a prospective study to propose valuable results.

## Declaration of competing interest

The author declares no competing interest.

## Data Availability

Data in this study will be available on reasonable request.

## Registration of research studies

UIN: researchregistry5813.
